# Long-Term Effects of Pre-Weaning Individual or Pair Housing of Dairy Heifer Calves on Subsequent Growth and Feed Efficiency

**DOI:** 10.3390/ani14050716

**Published:** 2024-02-24

**Authors:** Kaylee A. Riesgraf, Kent A. Weigel, Matthew S. Akins, Jennifer M. C. Van Os

**Affiliations:** 1 Department of Animal and Dairy Sciences, University of Wisconsin-Madison, Madison, WI 53706, USA; 2 USDA-ARS Institute for Environmentally Integrated Dairy Management, Marshfield, WI 54449, USA

**Keywords:** dairy cattle, calf housing, pair housing, animal welfare, neophobia, feed efficiency, methane emissions

## Abstract

**Simple Summary:**

As the dairy industry strives to improve the sustainability and efficiency of heifer rearing, it is important to understand the potential long-term impacts of early life events, such as social isolation stress, on the efficiency and performance of growing heifers. Pre-weaning pair housing has many immediate cognitive, growth, and feed intake benefits; however, little is known about the duration of these advantages over individually housed heifers. Pre-weaning isolation stress may have lasting adverse effects on heifer growth and feed efficiency, potentially inflating heifer rearing costs and decreasing farm profitability. To investigate possible long-term effects of pre-weaning housing, we measured the growth, feed efficiency, and methane emissions of 18-month-old heifers which had previously been paired or housed individually pre-weaning. Overall, pair-housed heifers maintained their initial body weight advantage over individually housed heifers with no adverse impacts on feed efficiency or methane emissions.

**Abstract:**

Our objective in this exploratory study was to evaluate the long-term impacts of pre-weaning social isolation vs. contact on subsequent growth and feed efficiency of Holstein heifers. As pre-weaned calves, 41 heifers were housed individually (*n* = 15 heifers) or in pairs (*n* = 13 pairs; 26 heifers). At 18 months of age, heifers were blocked by body weight and randomly assigned to one of three pens within a block (six to eight heifers per pen; six pens total), with original pairs maintained. Body weight (BW), hip height and width, and chest girth were measured at the start and end of the study. Each pen was given 3 days of access to a GreenFeed greenhouse gas emissions monitor to assess potential physiological differences between treatments in enteric methane emissions or behavioral differences in propensity to approach a novel object. During the 9-week study, heifers were fed a common diet containing 62.3% male-sterile corn silage, 36.0% haylage, 0.7% urea, and 1.0% mineral (DM basis). To calculate daily feed intake, as-fed weights and refusals were recorded for individual heifers using Calan gates. Feed samples were collected daily, composited by week, and dried to calculate dry matter intake (DMI). Feed refusal and fecal samples were collected on 3 consecutive days at 3 timepoints, composited by heifer, dried, and analyzed to calculate neutral detergent fiber (NDF), organic matter (OM), and DM digestibility. Feed efficiency was calculated as feed conversion efficiency (FCE; DMI/average daily gain [ADG]) and residual feed intake (RFI; observed DMI-predicted DMI). Paired and individually housed heifers did not differ in DMI, ADG, FCE, or RFI. Although no differences were found in initial or final hip height, hip width, or chest girth, heifers which had been pair-housed maintained a greater BW than individually housed heifers during the trial. Methane production, intensity, and yield were similar between treatments. Pre-weaning paired or individual housing did not impact the number of visits or latency to approach the GreenFeed; approximately 50% of heifers in each treatment visited the GreenFeed within 8 h of exposure. Digestibility of OM, DM, and NDF were also similar between housing treatments. In conclusion, pre-weaning pair housing had no adverse effects on growth, feed efficiency, or methane emissions at 18 to 20 months of age.

## 1. Introduction

Heifer rearing is a significant cost for dairy operations, as farmers invest in their replacement heifers for nearly two years but do not recoup this investment until after they become lactating cows and generate revenue. On average, feed comprises approximately 46% of rearing costs in the U.S. [1]. Costs of heifer rearing are greatest during the bred and pre-fresh periods, ranging from $2.80 to $3.20/head/day [1]. The challenge, therefore, is to sustainably minimize rearing costs by implementing management practices to improve the feed efficiency and animal welfare of dairy heifers, thereby also contributing to environmental and social sustainability. 

Substantial investments have been made in research toward improving feed efficiency in farm animals. Improving feed efficiency can increase land and water utilization efficiency and decrease greenhouse gas emissions, all of which can lead to the greater environmental sustainability of animal agriculture [2]. Enteric methane emissions accounted for 3.1% of total greenhouse gas emissions in the U.S. in 2021 [3]; however, the relationship between enteric methane emissions and feed efficiency is poorly understood. 

Feed conversion efficiency (FCE) is widely used in the swine, poultry, and, to a lesser extent, beef industries, where the ratio of feed consumption (typically dry matter intake, DMI) to average daily gain (ADG) in animals of similar age and body weight (BW) can be an effective measure of feed utilization efficiency. Beef cattle may vary in BW, and therefore net energy for maintenance (NEM), and lactating dairy cows vary widely in the amount of feed required to support net energy for lactation (NEL) while displaying positive or negative changes in BW (ΔBW). In such situations, one can use residual feed intake (RFI), the residual term from a model in which DMI is regressed on known energy sinks (BW, ΔBW, and NEL), as a measure of biological variation in feed utilization efficiency. Because RFI represents the difference between observed DMI and predicted DMI for an individual animal, a negative RFI value is preferred, as it signifies an efficient animal who consumes less feed than predicted. Recent research has focused on precision management [4,5] and genetic selection [6] as primary means to improve feed efficiency in lactating cows. Understanding feed efficiency in growing dairy heifers can help alleviate the expense of their rearing, but research regarding feed efficiency in this age class is limited, and thus work is needed to identify the factors which contribute to greater feed efficiency. 

Management practices during the pre-weaning period can impact the behavior, growth, and development of dairy heifers throughout the rearing period. Pre-weaned heifer calves are housed individually on approximately 70% of U.S. dairy farms [7], primarily due to the ease of feeding management and potentially reduced risk of disease transmission. However, there may be negative animal welfare, social sustainability, and calf performance consequences with individual housing, compared to full social contact. One study suggested pair or group housing is more acceptable to the public compared to individual housing [8], although this may depend on whether non-farming citizens are aware of cow–calf separation [9]. Social housing has been shown to be important for calf welfare based on studies which have demonstrated that calves prefer [10] and are motivated to gain social contact with conspecifics [11], and that socially housed calves show responses to ambiguous stimuli which are suggestive of optimism or positive emotional states [12]. Calves pair-housed early in life outperformed their individually housed counterparts when observed in cognitive tests [13,14]. Additionally, numerous studies, including those by our group [15], have shown equivalent or greater solid feed intake (DM basis), ADG, and weaning body weight in pair- or group-housed calves compared with individually housed ones (reviewed in [16]). These performance advantages appear to be driven, in part, by greater receptiveness of socially housed calves to novel situations and feeds [14,17,18,19] and reduced stress during weaning [20]. 

Earlier consumption of solids promotes rumen development [21]. Therefore, we hypothesize that early life pair housing may potentially result in longer-term impacts on feed efficiency and enteric methane emissions. However, few studies [22,23] have explored the long-term effects of social housing beyond the immediate post-weaning period, highlighting an important gap in the literature. The potential mechanism by which we speculate social calf housing could affect later-life performance, feed efficiency, and greenhouse gas emissions, is that early life social contact could accelerate intestinal microbiota maturation and subsequent gut development; however, this hypothesis was not directly addressed in the present study.

The primary objective of this exploratory study was to examine the long-term implications of pre-weaning paired versus individual housing on heifer growth and feed efficiency at 18 months of age. A secondary objective was to assess behavioral differences between early life housing treatments in heifers’ propensity to approach a novel object or feed source, along with potential physiological differences in enteric methane emissions. We hypothesized that the developmental benefits of pair housing during the pre-weaning period would positively affect the growth, performance, and feed efficiency and reduce neophobia in dairy heifers later in life. 

## 2. Materials and Methods

### 2.1. Design of Previous Pre-weaning Individual vs. Pair Housing Experiment

The present experiment was an exploratory follow-up study of Holstein heifers which were initially enrolled in a separate experiment [15] at the Emmons Blaine Dairy Cattle Research Center (Arlington, WI, USA) to study the effects of individual vs. pair housing on thermoregulatory and growth responses to seasonal cold stress in a continental climate. Heifers were randomly assigned to pair (*n* = 16 pairs, 32 calves) or individual (*n* = 16 calves) housing from birth through weaning. 

The original sample size was determined based on expected differences between individually and pair-housed calves for DMI of starter grain and ADG during the first 9 weeks of life. Previous studies comparing individually vs. pair-housed calves reported medium to very large (Cohen’s *d* ≥ 0.5 and ≥1.2) effect sizes for these outcomes [24,25,26]. A power analysis using those effect sizes indicated sample sizes of *n* = 5 to 16 experimental units (calf or pair of calves) would be required in each housing treatment.

During the pre-weaning period, all calves were raised in calf hutches (Calf-Tel Deluxe II, 1.1-m-wide × 2.1-m-long × 1.2-m-high inside dimensions; Hampel Corp., Germantown, WI, USA) at a ratio of 1 hutch per calf, with an outdoor area enclosed by wire fencing (1.2-m-wide × 1.8-m-long × 1.0-m-tall vs. 2.7-m-wide × 1.8-m-long × 1.2-m-tall for individually vs. pair-housed calves, respectively; this area was used to connect the 2 adjacent hutches in the latter treatment). 

All calves were fed pasteurized whole milk in the outdoor enclosure twice daily between 0400–0530 h and 1500–1600 h. All calves were initially hand-fed 1.9 L at each feeding by bottle (C14766; Nasco Education, Fort Atkinson, WI, USA). Once calves were determined by farm staff to be drinking sufficiently (at d 5 ± 1 of life, mean ± SD), milk volume was increased to 2.8 L per feeding, with the bottles hung on the fence. When calves reached d 14 ± 1 of life, milk allowance was increased to 3.8 L per feeding using larger bottles (Milk Bar Vitality System; The Coburn Company, Whitewater, WI, USA) hung from the fence. 

All calves were weaned in a step-down fashion based on age; for pair-housed calves, this was determined based on the younger calf in the pair. First, milk allowance decreased to 1.9 L twice daily at d 42 of life (average among all pair-housed calves: d 43 ± 1 of life), then to 1.9 L once daily (afternoon only) on d 49 of life (pairs: d 50 ± 1 of life). Milk was completely removed on d 53 of life (pairs: d 54 ± 1 of life). Calves remained in their hutches for at least 6 more d after weaning (9 ± 2 d), moving to post-weaning group housing no earlier than d 60 of life. 

In addition to milk, beginning on d 3 of life, calves were fed texturized starter (BSF 18; Vita Plus, Madison, WI, USA) composed of corn (43.5%), soybean meal (28.5%), cottonseed hull pellets (12.5%), molasses (5.0%), roasted soybeans (2.8%), wheat middlings (2.2%), calcium carbonate (1.6%), a mineral and vitamin premix (1.0%), dicalcium phosphate (0.6%), and salt (0.5%). Starter was fed in a plastic bucket (C18933; Nasco Education) inside each hutch, adjacent to the water pail. Starter was added twice daily (0600 and 1300 h) and fully replaced at least twice per week. To ensure ad libitum availability, the amount of starter offered was increased daily based on consumption (to achieve >5% refusals by weight). 

After weaning, calves remained in their original hutches until groups of 4 calves were available, at which time they were moved to group hutches (preserving pairs) until they were transported to the Marshfield Agricultural Research Station (Marshfield, WI, USA) at 93 ± 6 d of age, where they were housed in groups of 7 to 8 heifers. Heifers were then managed identically and fed the general herd heifer rations until the present study was initiated. 

Upon arrival at Marshfield, heifers were fed ad libitum dry grass hay and 3.2 kg of the same calf starter mix as previously fed, split into two feedings. Four weeks after arrival, heifers were transitioned from the grass hay/starter ration to a total mixed ration (TMR) over 4 weeks. Heifers were fed 4 different TMR diets during the rearing period, depending on heifer BW, with diets changing approximately every 90–100 kg BW. The initial TMR fed from 135 to 230 kg BW consisted of (% of diet DM): 60.8% haylage/baled silage, 33.5% ground shelled corn, 3.6% soybean meal, and 2.1% mineral/vitamin supplement (16.6% CP, 29.2% amylase neutral detergent fiber organic matter [aNDFom], 24.8% starch, and 2.82 Mcal metabolizable energy [ME]/kg DM). The second TMR fed from 230 to 320 kg BW consisted of 62.3% haylage/baled silage, 35.0% corn silage, 1.1% urea, and 1.6% mineral/vitamin supplement (15.6% CP, 47.5% aNDFom, 5.8% starch, and 2.24 Mcal ME/kg DM). The third TMR fed from 320 to 410 kg BW consisted of 68.1% haylage, 28.8% corn silage, 0.6% soybean meal, 0.9% urea, and 1.6% mineral/vitamin supplement (14.9% CP, 43.1% aNDFom, 13.2% starch, and 2.31 Mcal ME/kg DM). The fourth TMR fed to heifers >410 kg BW consisted of 68.6% haylage, 30.0% corn silage, 0.18% urea, and 1.2% mineral/vitamin supplement (15.6% CP, 39.5% aNDFom, 14.5% starch, and 2.42 Mcal ME/kg DM).

### 2.2. Design of Current Growth and Feed Efficiency Experiment 

During this study, the heifers were housed in 6 freestall pens with 8 mattress beds per pen. Forty-one of the initial 48 heifers (542 ± 16 d of age; *n* = 26 calves from 13 pairs; *n* = 15 individually housed calves) were retained for the present study. One individually housed and 3 pair-housed heifers were sold or died prior to the study; the hutch-mates of the latter 3 were excluded from enrollment to avoid potential confounding with the social stress associated with loss of their companion. 

All heifers were pregnant (mean ± SD; days carried calf = 120 ± 21) and blocked by initial BW (blocks 1 and 2: 560 ± 1.7 and 517 ± 13.6 kg, respectively, mean ± SD). Within blocks, heifers were randomly assigned to 1 of 3 pens, preserving pre-weaning pairs, with 6 to 8 heifers per pen (2–3 individually housed and 2–3 pair-housed heifers per pen). Next, heifers within each pen were randomly assigned to Calan gates (American Calan; Northwood, NH, USA) accessed using neck collar transponders, which allowed for measurement of individual feed intake. For habituation, heifers were moved into their respective pens 2 weeks prior to the initiation of data collection and provided with feed at the open bunk for 4 d. Next, the Calan gates were lowered into place, and all heifers had access to all gates for of 3 d. Finally, heifers were trained to use their specific gates during the final 7 d of the pre-trial period; all heifers were trained successfully by the end of this acclimation week.

### 2.3. Body Measurements and Feed Efficiency Phenotypes

Body weight was measured on d −2, −1, 0 and 61, 62, 63 of the experiment, where d 1 and 63 represent the first and last dates of daily feed intake measurement, respectively; the 3 measurements at the start and end of the study were averaged to represent an initial and final weight per heifer. Hip height, hip width, and chest girth measurements were taken on d 0 and 63. Average daily gain was calculated by subtracting initial BW from final BW and dividing by 63 d, and FCE was determined by dividing average DMI by ADG. Residual feed intake was calculated as the residual of the regression of DMI on mid-point metabolic BW (MBW) and ADG:

y_i_ = β_0_ + β_1_ × MBW_i_ + β_2_ × ADG_i_ + e_i_ (1)

where y_i_ is the daily DMI of the i^th^ heifer, β_0_ is the intercept, β_1_ is the partial regression coefficient of DMI on MBW (−0.12), β_2_ is the partial regression coefficient of DMI on ADG (1.98), and e_i_ is the random error term which represents RFI. Because RFI is calculated as observed DMI minus expected DMI, a negative RFI value indicates a more efficient animal.

### 2.4. Diet and Feed Samples

Heifers were fed a TMR composed of 62.7% male-sterile corn silage, 36.0% alfalfa-grass haylage, 0.5% urea, and 0.8% mineral on a DM basis throughout the trial (Table 1). During week 5 of the trial, a switch in haylage source resulted in a decrease in neutral detergent fiber (NDF, % of DM) and an increase in total digestible nutrients (TDN, % of DM). This higher quality haylage was fed for weeks 5 to 9 of the trial. At 1000 h daily, refusals were removed from the Calan gates and weighed, and a fresh TMR was delivered. Single TMR and refusal samples were collected daily and composited by week, and diet components were sampled weekly. Dry matter was calculated by drying samples at 55 °C for 48 h in a forced air oven. Samples were ground to pass a 1 mm screen (Wiley Mill, Arthur H. Thomas, Philadelphia, PA, USA) and analyzed by a commercial laboratory using a wet chemistry package (Dairyland Labs, Arcadia, WI, USA; Table 1).

### 2.5. Fecal Samples and Total-Tract Digestibility

Individual TMR and fecal samples were taken once daily on d 54, 55, and 56 to determine ration total-tract digestibility. Fecal samples were taken thrice daily (0800, 1200, and 1600 h) to account for diurnal variability and composited by heifer. The TMR and fecal samples were dried at 55 °C for 48 h in a forced air oven, ground to pass a 1 mm screen (Wiley Mill, Arthur H. Thomas, Philadelphia, PA, USA) and analyzed for ash (combusted at 500 °C for 6 h), NDF [27], and undigested NDF at 240 h (uNDFom240 [28]).

### 2.6. Enteric Greenhouse Gas Emissions

A GreenFeed (C-Lock; Rapid City, SD, USA) greenhouse gas emissions monitor mobile unit was used to measure methane (CH_4_) and carbon dioxide (CO_2_) from the exhalant of individual heifers. The GreenFeed detects cattle head proximity, reads a radio frequency identification tag, and activates a fan to pull air and exhalant through the sampling hood and incorporated gas measurement equipment. Heifers visited the GreenFeed voluntarily, enticed by alfalfa pellets (95.2% DM; 42.0% NDF, 18.7% CP, 56.8% TDN on a DM basis). Each heifer received up to 6 deliveries of alfalfa pellets (35 g) in 40 s intervals at each visit, and the GreenFeed automatically recorded the timestamp of each visit. Heifers also had free access to the TMR at their respective Calan gates throughout the GreenFeed measurement period.

Pens 1–6 had access to the GreenFeed for 24 h/d on d 41–44, 44–47, 47–50, 50–53, 53–56, and 56–59, respectively. Heifers had no previous experience or training with the GreenFeed, although heifers in the latter pens could watch those in earlier pens using it; therefore, the order in which pens had access to the GreenFeed was recorded to account for possible bias in GreenFeed usage due to social facilitation. When the GreenFeed was moved to a new pen, it was calibrated for CO_2_ and CH_4_, and heifers were given access within 1 h.

Methane production (g/d) and CO_2_ production (g/d) were calculated as 3 d averages of emissions of individual heifers. Methane intensity (g/kg ADG) and methane yield (g/kg DMI) were calculated using 3 d averages for methane production and calculated ADG values and DMI means from the 63 d trial period. Only heifers who visited the GreenFeed were included in the calculation and analysis of methane production, CO_2_ production, methane intensity, and methane yield (*n* = 17 paired; *n* = 9 individually housed). The number of GreenFeed visits and the latency to first visit the GreenFeed were recorded to evaluate potential behavioral differences between individually and pair-housed calves, as the GreenFeed was a novel object to these heifers. Heifers who did not visit the GreenFeed were assigned a value of zero visits. Latency phenotypes were calculated as the elapsed time between the end of the calibration period in a given heifer’s pen (i.e., when the GreenFeed became available) and the heifer’s first visit to the GreenFeed. Latency phenotypes of heifers who did not visit the GreenFeed during the 3 d measurement period were considered as right-censored (i.e., unobserved and thus >4320 min).

### 2.7. Data Analysis

Two heifers (both pair-housed, not hutch-mates) were excluded from the statistical analysis due to illnesses during the last two weeks of the measurement period, which caused significant reductions in feed intake and low final body weights. Data were analyzed using the *lme4*, *lmerTest*, and *lsmeans* packages of the R statistical software, version 4.1.1 (R Core Team, 2021). Models were determined using sequential model selection based on Akaike information criterion; the variable for days carried calf was initially considered but not included in the final model. The experiment was a randomized complete block design with 2 weight blocks and with heifer as the experimental unit. All models included pen nested within block as a random effect. Effects were considered significant when *p* < 0.05, and tendencies were defined as 0.05 ≤ *p* < 0.10.

Body weight and body measurements (hip height, hip width, chest girth) were compared between treatments, separately at the initial and final measurement timepoints, using linear mixed models with treatment and block as fixed effects. The model for DMI (averaged over the 63 d trial and including any pellet intake on a DM-basis during the 3-day GreenFeed measurement period) included treatment, ADG, MBW, and block as fixed effects. In addition, weekly DMI data were analyzed using fixed effects of treatment, week, the treatment × week interaction, and block.

The models to compare the treatments for ADG, FCE, RFI, methane intensity, methane yield, and the digestibility variables included treatment and block as fixed effects. The models for methane and CO_2_ production included the additional fixed effects of average daily DMI and midpoint BW. The probability of visiting the GreenFeed (binary outcome; logistic regression) and the number of GreenFeed visits (count) were modeled using treatment, block, and order of GreenFeed unit exposure as fixed effects. Latency to first visit the GreenFeed was analyzed using Kaplan–Meier model survival analysis using treatment, block, and order of pen exposure as fixed effects.

## 3. Results

### 3.1. Heifer Growth and Feed Efficiency

Pair- and individually housed heifers did not differ in hip height or hip width at the start or end of the trial (*p* ≥ 0.33, Table 2). Initial chest girth was greater in the pair-housed heifers than the individually housed ones (*p* = 0.01), but final chest girth did not differ between treatments (*p* = 0.13), perhaps due to numerically greater ADG among individually housed heifers during the study period. Pair-housed heifers weighed more than individually housed heifers at the start of the trial (*p* = 0.03), with some limited evidence to suggest this difference was maintained at the end of the study (*p* = 0.05). No differences between treatments were observed in growth, intake, or efficiency, as measured by ADG, total daily DMI, FCE, and RFI, respectively (*p* ≥ 0.33). No treatment differences were detected in GreenFeed pellet intake during the 3-day measurement period (*p* = 0.16).

**Figure 1:**
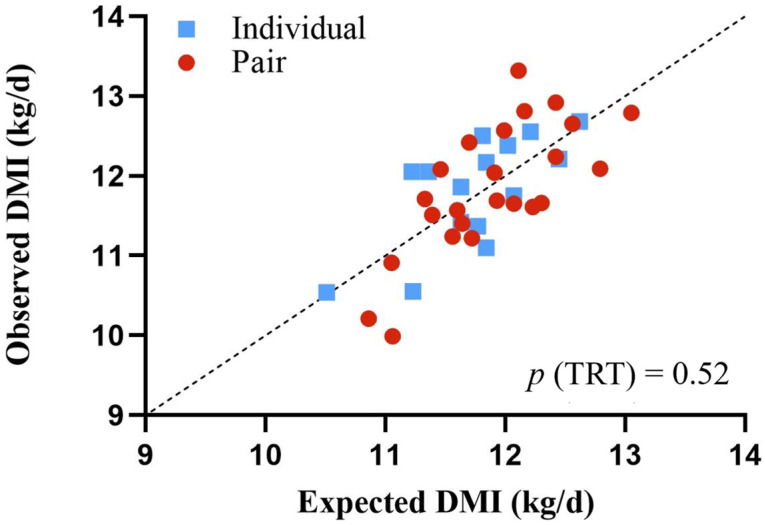
Residual feed intake during 18 to 20 months of age for dairy heifers previously housed in pairs (red dot, •; *n* = 24) or individually (blue square, ■; *n* = 15) from birth through weaning. Paired heifers were housed with two calves sharing adjoined calf hutches and an outdoor run space, and individual heifers were housed with a single calf per hutch and outdoor run space. Efficient animals with observed intakes less than expected intakes are below the dotted line (negative RFI), while inefficient animals with observed intakes greater than expected intakes are above the line (positive RFI).

Figure 1 illustrates the concept of RFI, in which efficient animals with observed intakes less than expected intakes are below the line (negative RFI), whereas inefficient animals with observed intakes greater than expected intakes are above the line (positive RFI).

**Figure 2:**
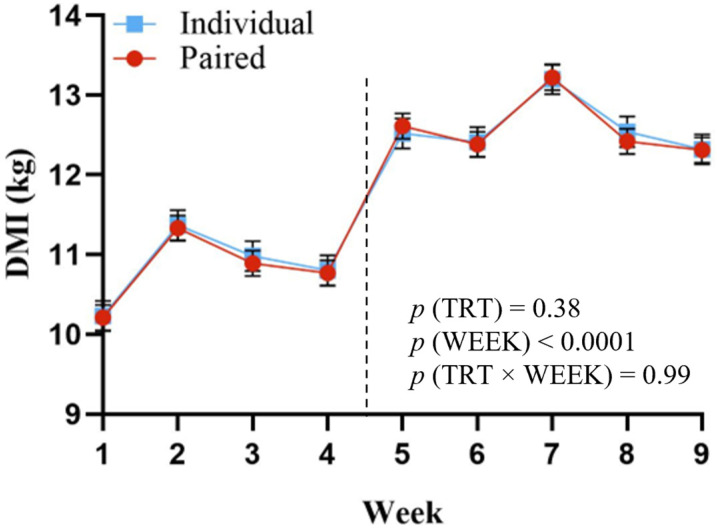
Weekly DMI from 18 to 20 months of age for dairy heifers previously housed in pairs (red dot, •; *n* = 24) or individually (blue square, ■; *n* = 15) from birth through weaning. Paired heifers were housed with two calves sharing adjoined calf hutches and outdoor run space, and individual heifers were housed with a single calf per hutch and outdoor run space. The vertical dashed line indicates a change in haylage source between weeks 4 and 5 of the study.

Weekly DMI results are shown in Figure 2. There were no treatment differences in weekly DMI (*p* = 0.38, Figure 2), but values increased in both treatment groups in week 5, likely due to a change in haylage source.

### 3.2. Enteric Methane Emissions and GreenFeed Visits

Emissions data from individually (*n* = 9 of 15) or pair-housed (*n* = 17 of 24) heifers which visited the GreenFeed at least once were included in methane production, CO_2_ production, and methane yield calculations shown in Table 3. All animals were included in the analysis of GreenFeed visits, and heifers that did not visit were assigned a value of zero. There were no differences in methane production, CO_2_ production, methane yield, methane intensity, or the number of GreenFeed visits between treatments (*p* ≥ 0.21).

**Figure 3:**
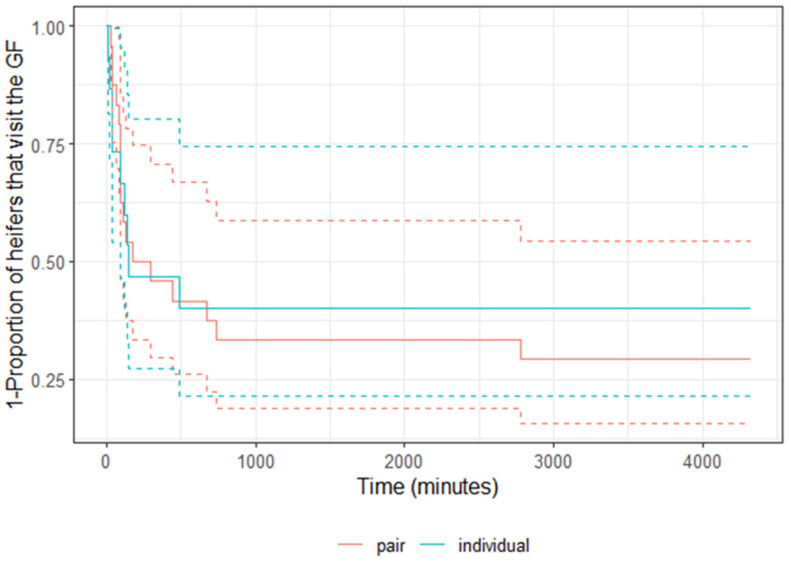
Survival curve of latency to first visit the GreenFeed unit at 20 months of age for dairy heifers previously housed in pairs (red line; *n* = 24) or individually (blue line; *n* = 15) during the pre-weaning period. From birth to weaning, paired heifers were housed with two calves sharing adjoined calf hutches and an outdoor run space, and individual heifers were housed with a single calf per hutch and outdoor run space. Solid lines indicate the model estimates with dashed lines representing the 95% confidence intervals.

Despite slight numerical differences (pair- vs. individually housed heifers: 17 out of 24 vs. 9 out of 15), when accounting for the order of pen exposure to the GreenFeed (*p* = 0.28), there was no treatment difference in the probability of approaching the GreenFeed during the observation period (*p* = 0.44). Likewise, the latency for heifers to first approach the GreenFeed did not differ between treatments (*p* = 0.70); 50% of the heifers in each treatment visited the GreenFeed within 8 h (500 min) of exposure (Figure 3). The shortest latency values among individually vs. pair-housed heifers were 14 vs. 26 min, respectively.

### 3.3. Nutrient Intake, Fecal Output, and Digestibility

Results regarding nutrient intake, fecal output, and digestibility of DM, OM, and NDF are presented in Table 4. During the digestibility sampling period, heifers did not differ in the intake, output, or digestibility of DM, OM, or NDF (*p* ≥ 0.50).

## 4. Discussion

Feed is typically the most expensive input on a dairy operation, and it is important to understand how socially sustainable management practices, such as the strategy of pair housing calves during the pre-weaning period, may impact subsequent feed efficiency. To our knowledge, this is the first study to evaluate the long-term effects of pre-weaning individual versus pair housing in pregnant dairy heifers. This exploratory study leveraged heifers which were previously enrolled in a separate experiment on outcomes during early life pair vs. individual housing. Therefore, the sample size in the present study was constrained by the number of heifers remaining from the independent study which ended 16 months earlier, and this limitation impacted our statistical power. Overall, we found that individually and pair-housed heifers performed similarly in growth, feed efficiency, and enteric methane emissions during the study period. Pair-housed heifers had an initial BW advantage which they appeared to maintain during the study, but there was no other evidence to support persistent positive or negative effects of individual versus pair housing during the pre-weaning period on intake, growth, efficiency, or emissions outcomes later in life.

### 4.1. Heifer Growth and Feed Efficiency

The social housing of pre-weaned calves, compared to the U.S. industry status quo of individual housing, may benefit the social sustainability of dairy farming [8]. Pair or group housing has been documented to improve calf welfare and emotional states [12] by addressing a preference [10] and motivation [11] for social contact with similar-age conspecifics. Furthermore, this practice has been shown to increase learning ability [13,14] and resilience to weaning stress [20], as well as calf growth performance, including greater ADG, grain intake, and BW relative to individually housed calves [16]. Indeed, no published studies to date have reported reduced BW or ADG in social groups compared to individual housing. In a review [16] of 16 studies comparing the growth and performance of calves housed individually, in pairs, or groups of up to six calves during or immediately following weaning, all reviewed studies reported either positive or no detectable impacts on BW, ADG, and grain intake in favor of paired or group-housed calves. Since 2016, other studies have reported similar findings of greater solid food consumption from birth through weaning and less phobia of novel feeds, but no difference in BW or ADG [15,19,29,30]; however, all measurements in these studies were taken in the first 14 weeks of life. It remains unclear why some studies find performance advantages with social housing, whereas others do not detect differences; dietary factors, such as milk allowance [21] or the composition of solid feeds (e.g., concentrate vs. TMR [31]), likely play a role.

To our knowledge, only two previous studies to date have compared long-term growth or production outcomes between formerly pair- vs. individually housed calves. In one U.S. study [22] at 16 weeks of age, after calves were moved to post-weaning group housing, no effects of previous individual vs. pair housing were found on BW or lifetime ADG. In a recent U.K. study [23] tracking the daily liveweight gain (DLWG) of calves on a commercial dairy farm through confirmation of pregnancy, the heifers formerly housed individually tended to have greater DLWG than those housed in pairs, but birthweight, birth season, and pre-weaning disease were significant factors affecting growth. Interestingly, that study found that formerly pair-housed calves tended to produce approximately 12% more total milk during their first lactation compared to their individually housed counterparts, perhaps because the latter had a greater hazard of exiting the herd. The finding by those authors highlights a continued gap in the literature about the long-term health impacts of individual vs. social housing, which could in turn affect productivity and farm profitability. In the current study, in which heifers averaged 77 weeks of age at the beginning of the trial, pair-housed heifers were significantly larger in chest girth and BW on day 0 of the study, with some evidence that the latter advantage was maintained during the study period. Under the conditions of this study, however, no other lasting effects of pre-weaning individual or pair housing on mature heifer growth and performance were detected.

To improve the economic and environmental sustainability of dairy heifer rearing, it is imperative to understand why some animals are more efficient than others. Our study is the first to evaluate RFI in the context of previous individual vs. social calf housing. Gravid heifers of similar age and weight to those included in the current trial were studied previously [32,33] and subjected to either control (ad libitum) or limit-fed diets. Heifers in the control groups in those experiments exhibited similar growth, DMI, ADG, and FCE compared with heifers in the current study, suggesting our results are consistent with the scientific literature to date. In our group’s other previous work [34,35], heifers were fed diets diluted by high-fiber, low-energy forages, with similar ADG in the treatment and low efficiency groups, respectively, compared with those observed in the current study; however, DMI and FCE reported in those studies were lower than in the current study. While such dietary manipulations may influence feed intake and feed efficiency in yearling heifers, our results suggest that differences between the present study and the earlier ones are unrelated to housing strategies during the pre-weaning period. Nonetheless, few studies have investigated the effects of pre-weaning social contact on feed efficiency, and further work is needed to draw more definitive conclusions. Future studies could also investigate the impacts of early life social housing on later life reproductive outcomes, which also affect the animals’ productive longevity and the economic sustainability of dairy farming, as well as more explicitly quantify the economic impacts of converting from individual to social calf housing.

### 4.2. Behavioral Response to GreenFeed and Enteric Methane Emissions

Several previous studies [17,18,19] have reported that calves housed in pairs or groups approach novel objects and feedstuffs more readily than their individually housed counterparts. The GreenFeed used to collect enteric emissions data in the present was novel to the heifers and provided an opportunity to examine their responses at a more advanced age than previously evaluated in the literature. Despite numerical trends, we did not detect statistical differences between individually and pair-housed treatments in the odds of visiting the GreenFeed or the latency to first visit the machine, after accounting for order of exposure. Because latter pens had visual exposure to the GreenFeed, however, the effect of novelty may have been reduced, thereby affecting our ability to detect treatment differences. Regardless of pre-weaning housing treatment, 50% of heifers in our study visited the GreenFeed within 500 min of exposure, and individual latencies as short as 14 min were observed.

However, about a third of the heifers never visited the GreenFeed, suggesting our exposure period of 3 days may not have allowed adequate time for all heifers to overcome their fears of novelty [36]. In a separate recent study by our group, carried out at the same facility using different dairy heifers of similar age, only 13% of heifers failed to visit the GreenFeed after 8 days of exposure (K.A.R., unpublished data). Another consideration is that heifers in the current study were exposed to the GreenFeed as a group, whereas in some previous studies, calves were tested individually for neophobia responses. Additional studies are needed to fully understand the long-term behavioral impacts of pre-weaning individual versus pair housing on responses to novelty.

In addition to evaluating heifers’ responses to the novel GreenFeed, we sought to add data to the literature on greenhouse gas emissions of growing dairy heifers. According to the U.S. Environmental Protection Agency [3], agriculture accounted for 9.3% of 2021 total U.S. greenhouse gas emissions, of which 32.6% can be attributed to enteric fermentation. Potential benefits of improving feed efficiency include decreasing enteric methane emissions and improving land use efficiency for food production [2]. In the present study, we hypothesized that both feed efficiency and enteric methane emissions may be influenced by gut development, which could in turn be shaped by early life solid intake patterns. Greater solid feed intake has been previously documented in pair-housed calves compared to individually housed ones [16], including some limited evidence we observed during week 9 of life [15] among the calves which were later enrolled into the present study. Future studies could more directly evaluate these potential causal mechanisms linking early life housing, gut development, feeding patterns and efficiency, and enteric methane emissions. Such future work incorporating innovative physiological approaches could expand our understanding of calf development and welfare.

In the present study, methane production, CO_2_ production, and methane yield results were similar to those reported in beef heifers [37], when CH_4_ and CO_2_ flux were measured with a GreenFeed on 1099 beef steers, heifers, and bulls ranging from 368 to 910 days of age, which were fed a TMR of 23.5% hay and 76.5% concentrates on a DM basis. In another study [36] in which 21-month-old Charolais heifers were measured with a GreenFeed for 8 weeks, the reported methane production and CO_2_ production values were much greater than those of the present study, perhaps due to a shorter measurement period or greater average BW, as compared with the current study. However, their reported methane yield values were similar to methane yield results of the current study, suggesting that observed differences in methane production may have been due to differences in feed intake. In the present study, there were no differences in CH_4_ or CO_2_ emissions between paired or individually housed heifers, perhaps due to similar ADG and DMI between treatments. The lack of treatment differences in both feed efficiency and greenhouse gas emissions in the current study suggests a practical implication that pre-weaning social housing may have neutral effects on environmental sustainability. However, a study limitation is that measurement time with the GreenFeed was limited to 3 days per pen due to availability of the GreenFeed equipment, whereas other researchers [36,38] have recommended a measurement period of at least 14 days to minimize within-animal variation. Although an extended measurement period may have improved the precision of our CH_4_ and CO_2_ phenotypes, our results suggest that enteric emissions may not be significantly impacted by pre-weaning housing strategies. Nonetheless, because not all heifers in our study visited the GreenFeed, caution must be used in interpreting the lack of treatment differences, and future work is needed to draw more definitive inferences. In addition, longer measurement periods could allow for more detailed study of diurnal patterns in emissions.

### 4.3. Nutrient Digestibility

Variation in the rumen microbiota between ruminants can impact forage digestibility and, potentially, feed efficiency. Previous studies [39,40] reported greater NDF digestibility in low RFI (more efficient) cows compared with their high-RFI contemporaries, perhaps due to longer rumen retention time and decreased DMI. In the current study, we found no differences in DM, OM, or NDF digestibility of formerly paired or individually housed heifers. Indeed, all heifers in the current study showed similar RFI, methane production, and methane yield, which may reflect the lack of treatment differences in diet digestibility. Results of the current study are consistent with other published literature. For example, in a previous study by our group [41], heifers were fed a control diet similar to that of the current study, and results for DM, OM, and NDF intake, output, and digestibility were similar. Furthermore, another study at the same facility [35] reported similar DM, OM, and NDF digestibility results to those of the current study and found no differences in digestibility between heifers with high or low genomic predisposition for RFI. However, no other studies to date have investigated the effects of pre-weaning housing strategy on nutrient digestibility and efficiency later in life, and this topic continues to represent an area for further study.

## 5. Conclusions

Eighteen-month-old dairy heifers which were pair-housed pre-weaning weighed more than their individually housed counterparts at the beginning of the study, with some evidence that they maintained this advantage during the two-month trial. However, within the conditions of this study, previous individual versus pair housing of calves during the pre-weaning period did not impact other body measurements, growth rate, feed intake, feed efficiency, or enteric methane emissions between 18 to 20 months of age. Previous studies of the differences in outcomes between calves housed individually or in pairs during the pre-weaning period have reported growth advantages for the latter calves up to 14 weeks of age. Further studies are needed to assess the long-term effects of the individual, pair, or group housing of dairy calves and to document if and when compensatory growth of individually housed calves may occur. Larger sample sizes during the initial calf experiments are recommended for future longitudinal studies of this type to ensure that adequate statistical power remains when accounting for attrition and unexpected potential confounding factors such as illness. That said, results of the present exploratory study seem to indicate that the pair housing of dairy calves may enhance the social sustainability of dairy farming with no adverse impacts on heifer performance or farm profitability.

## Figures and Tables

**Table 1:** Ingredient composition of diet and mean nutrient composition of TMR and individual diet components based on weekly analysis.

		Dietary Component	
Item	Diet	Corn Silage	Haylage
Ingredient, % of DM			
Male-sterile corn silage	62.7	–	–
Haylage	36.0	–	–
Urea	0.50	–	–
Mineral supplement	0.80	–	–
Nutrient composition (DM basis)			
DM, %	45.2	36.2	54.4
CP, %	13.4	9.87	13.0
aNDF, %	56.9	57.6	57.5
aNDFom, %	55.5	56.3	54.8
NDFD48, % of NDF ^1^	66.2	63.8	68.7
Starch, %	1.18	3.78	0.36
Ash, %	7.82	4.94	8.46
Ca, %	0.54	0.30	0.61
P, %	0.31	0.3	0.31
Mg, %	0.28	0.23	0.26
K, %	2.44	1.35	2.94
NFC, %	22.9	28.6	22.8
Energy estimate ^2^			
TDN, %	56.2	63.2	57.8
NEG, Mcal/kg DM	0.65	0.82	0.7
NEM, Mcal/kg DM	1.21	1.41	1.27
ME, Mcal/kg DM	2.07	2.27	2.13

^1^ In vitro NDF digestibility after 48 h incubation. ^2^ Based on NASEM 2001 calculations.

**Table 2:** Performance of 18- to 20-month-old dairy heifers previously housed individually or in pairs during the pre-weaning period (LSM ± SEM).

	Treatment ^1^		
Item	Paired (*n* = 24)	Individual (*n* = 15)	*p*-Value
Initial			
Body weight, kg	555 ± 5.7	535 ± 7.1	0.03
Hip height, cm	144 ± 0.5	145 ± 0.6	0.51
Hip width, cm	52.3 ± 0.6	51.8 ± 0.7	0.41
Chest girth, cm	192 ± 0.8	189 ± 1.0	0.01
Final			
BW, kg	614 ± 5.6	597 ± 6.9	0.05
Hip height, cm	145 ± 0.6	145 ± 0.7	0.80
Hip width, cm	52.4 ± 0.5	51.9 ± 0.6	0.33
Chest girth, cm	199 ± 0.9	197 ± 1.1	0.13
Feed Efficiency ^2^			
Total DMI, kg/d	11.8 ± 0.1	11.9 ± 0.1	0.40
GreenFeed pellet intake, DM kg/d	0.04 ± 0.1	0.03 ± 0.1	0.16
ΔBW, kg	59.5 ± 2.5	62.0 ± 3.0	0.49
ADG, kg/d	0.94 ± 0.04	0.98 ± 0.05	0.52
FCE, kg DMI/kg ADG	13.1 ± 0.6	12.3 ± 0.7	0.33
RFI, kg/d	−0.04 ± 0.11	0.06 ± 0.13	0.56

^1^ Paired: from birth to weaning, heifers were housed with two calves sharing adjoined calf hutches and outdoor run space. Individual: from birth to weaning, heifers were housed with a single calf per hutch and outdoor run space. ^2^ FCE = feed conversion efficiency; ADG = average daily gain; RFI = residual feed intake. The calculations for total DMI, FCE, and RFI include pellet consumption from the GreenFeed machine.

**Table 3:** Enteric methane and carbon dioxide emissions of 20-month-old dairy heifers previously housed individually or in pairs during the pre-weaning period (LSM ± SEM).

	Treatment ^1^		
Item	Paired (*n* = 17)	Individual (*n* = 9)	*p*-Value
Methane production, g CH_4_/d	262 ± 5.9	262 ± 8.8	0.95
Carbon dioxide production, g CO_2_/d	8290 ± 87.5	8347 ± 124.5	0.70
Methane yield, g CH_4_/kg DMI	22.4± 0.61	21.9 ± 0.87	0.60
Methane intensity, g CH_4_/kg ADG ^2^	286 ± 18.1	264 ± 22.7	0.35
GreenFeed visits per day ^3^	13.7 ± 2.07	8.7 ± 2.59	0.21

^1^ Paired: from birth to weaning, heifers were housed with two calves sharing adjoined calf hutches and outdoor run space. Individual: from birth to weaning, heifers were housed with a single calf per hutch and outdoor run space. ^2^ ADG = average daily gain. ^3^ Although emissions are reported only for those heifers who visited the GreenFeed (*n* = 17 pair; *n* = 9 individual), all heifers in the study (*n* = 24 pair, after excluding 2 due to health issues; *n* = 15 individual) were included in the calculation of visits per day, with a value of zero assigned to heifers with no visits.

**Table 4:** Nutrient digestibility of 20-month-old dairy heifers previously housed individually or in pairs during the pre-weaning period (LSM ± SEM).

	Treatment *		
Item	Paired (*n* = 24)	Individual (*n* = 15)	*p*-Value
Nutrient intake, kg/d			
DM	11.9 ± 0.14	11.7 ± 0.18	0.52
OM	10.9 ± 0.18	11.0 ± 0.22	0.75
NDF	6.57 ± 0.11	6.63 ± 0.13	0.72
Fecal output, kg/d			
DM	4.88 ± 0.10	4.96 ± 0.12	0.59
OM	4.32 ± 0.08	4.40 ± 0.10	0.54
NDF	2.71 ± 0.06	2.76 ± 0.07	0.60
Digestibility, %			
DM	58.8 ± 0.45	58.5 ± 0.56	0.62
OM	60.5 ± 0.39	60.1 ± 0.49	0.50
NDF	58.7 ± 0.44	58.3 ± 0.55	0.59

* Paired: from birth to weaning, heifers were housed with two calves sharing adjoined calf hutches and outdoor run space. Individual: from birth to weaning, heifers were housed with a single calf per hutch and outdoor run space.

## Data Availability

The data presented in this study are available on request from the corresponding author.
